# AIMP1-Derived Peptide Secreted from Hair Follicle Stem Cells Promotes Hair Growth by Activating Dermal Papilla Cells

**DOI:** 10.7150/ijbs.101127

**Published:** 2024-10-21

**Authors:** YounHa Kim, Sang Bum Kim, Ho Lee, Doyeun Kim, Soon Sun Bak, Ina Yoon, Seongmin Cho, Seung Jae Jeong, Yoon Jeon, Jina Kim, Ji-hee Kim, Soohwan Oh, Khas-Erdene Battogtokh, Min Chul Park, Young Kwan Sung, Sunghoon Kim

**Affiliations:** 1 Department of Integrative Biotechnology, Interdisciplinary Graduate Program, College of Pharmacy, Medicinal Bioconver-gence Research Center, Institute for Artificial Intelligence and Biomedical Research, Gangnam Severance Hospital, Yonsei University, 85 Songdogwahak-ro, Yeonsu-gu, Incheon 21983, South Korea.; 2 Graduate School of Cancer Science and Policy, National Cancer Center, Gyeonggi 10408, South Korea.; 3 Department of Immunology, School of Medicine, Kyungpook National University, Daegu 41944, South Korea.; 4 College of Pharmacy, Sahmyook University, Seoul, South Korea.; 5 CureBio Therapeutics Co., Ltd, 12fl, 91, Changnyong-daero 256beon-gil, Yeongtong-gu, Suwon-si, Gyeonggi-do, South Korea.; 6 College of Pharmacy, Korea University, 2511 Sejong-ro, Sejong 30019, South Korea.; 7 College of Pharmacy and Inje institute of pharmaceutical sciences and research, Inje university, Gimhae, South Korea.; 8 Yonsei Institute of Pharmaceutical Sciences, College of Pharmacy, Yonsei University, Incheon, 21983, South Korea.

**Keywords:** hair follicle stem cell, dermal papilla cell, AIMP1, hair growth

## Abstract

Hair follicle stem cells (HFSCs) and dermal papilla cells (DPCs) are crucial in the biogenesis and maintenance of hair follicles (HFs). This study demonstrated that a fragment derived from aminoacyl-tRNA synthetase-interacting multifunctional protein1 (AIMP1) secreted from HFSCs activated DPCs and maintained HF homeostasis. A histological analysis revealed that AIMP1 levels in HF decreased with hair loss. Hair regrowth in AIMP1-induced mice was faster than in non-induced mice. Deletion mapping revealed 41 amino acids (TN41, aa 6-46) as the active region of AIMP1. The N-terminal peptide fragment of AIMP1 generated by MMP1 was secreted from Wnt-treated HFSCs to activate DPCs. TN41 activated Akt and ERK, increased β-catenin, and enhanced DPC activation. TN41 promoted hair shaft elongation in cultured human HFs and improved the hair-inducing activity of cultured DPC spheroids. Our findings suggest that the AIMP1 fragment secreted from HFSCs stimulates active hair regrowth through activating DPCs.

## Introduction

Hair follicles (HFs) undergo cyclical transformations through the stages of growth (anagen), regression (catagen), and rest (telogen). HFs produce hair shafts during the anagen phase, while during the catagen and telogen phases, HFs reset and prepare hair follicle stem cells (HFSCs) and dermal papilla cells (DPCs). HFSCs and DPCs communicate via signaling molecules such as Wnt7b, FGF7, and Noggin to initiate the next growth phase and create a new hair shaft for hair homeostasis 1.

HFSCs, which reside in the bulge area of the HF, sustain cyclic hair regrowth over repeated cycles 2. Communication between HFSCs and their niches is reciprocal since stem cells regulate homeostasis and maintenance of niches 3. Mouse HFSCs do not display apparent decreases in numbers 4; however, aging causes imbalanced cytokine signaling with their niches and diminished colony-forming capability 5. Loss of communication between HFSCs and their niches decreases hair density and increases hair thinning in mammals that live relatively long periods 6.

DPCs trigger hair cycling via a paracrine signaling mechanism 7. Specifically, crosstalk between DPCs and HFSCs is essential for ensuring postnatal hair growth and HF cycling 8. DPCs are physically closest to HFSCs during telogen, and various factors secreted by these cells are likely involved in the progression from telogen to anagen. Understanding the mechanisms of this telogen-to-anagen transition and anagen maintenance is critical to ensuring successful hair loss treatment.

Aminoacyl-tRNA synthetase-interacting multifunctional protein1 (AIMP1), initially identified as a member of the mammalian multi-tRNA synthetase complex 9, is secreted in response to cytokine stimulation and exerts distinct extracellular activities depending on the target cells 10-14.

Human AIMP1 mutations are associated with severe neurodegenerative disorders. Mutations in AIMP1 have been linked to the development and maintenance of axon cytoskeleton integrity and regulation of neurofilaments 15, 16. Furthermore, AIMP1 is associated with cellular homeostasis. Macrophages secrete AIMP1 following stimulation with tumor necrosis factor (TNF)-α to enhance wound healing, a process mediated by AIMP1-induced fibroblast proliferation and collagen synthesis via Akt and ERK activation 17. Deletion-mapping analysis revealed that the N-terminal domain of AIMP1 stimulates fibroblast and mesenchymal stem cell (MSC) proliferation 18, 19. Additionally, the expression of AIMP1 is high in DPCs and dermal sheaths, suggesting a novel role for AIMP1 in the skin or HFs 20.

In this study, we investigated the role of AIMP1 in HF maintenance using aged mice and AIMP1-transgenic and deletion mouse models and assessed the function of AIMP1 in the crosstalk between DPCs and HFSCs, confirming the efficacy of AIMP1 secreted by HFSCs in promoting DPC activity. Furthermore, we identified the specific active domain of AIMP1 responsible for this effect. Our findings indicate that AIMP1 regulates hair growth through its interaction with DPCs and suggests that AIMP1, a component of the multi-tRNA synthetase complex, and its active domain, TN41, are potential therapeutic candidates for the treatment of hair loss.

## Materials and methods

Please see Supplementary Materials and Methods for more information.

### Generation of inducible hAIMP1 mice

To generate an inducible hAIMP1 knock-in mouse, the tetO-hAIMP1 construct was cloned to contain human AIMP1 cDNA under the control of a minimal promoter from hCMV fused to the tetO sequence.

This construct was subcloned into a ROSA targeting vector (Soriano P's lab, NY, USA). The targeting vector was electroporated into mouse embryonic stem (ES) cells (E14TG2a). Correctly targeted clones were injected into C57BL/6 blastocysts for chimera generation. TRE-hAIMP1 mice were crossed to CAG-rtTA3 (Jackson Laboratories, Bar Harbor, ME), KRT14-rtTA (Jackson Laboratories), or KRT14-cre (Jackson Laboratories) with Rosa26-CAGs-LSL-rtTA3 (Jackson Laboratories) to generate systemic or skin-specific hAIMP1-inducible mice.

### Immunofluorescence staining

Paraffin sections were used for immunofluorescence (IF) analysis. Slides were incubated for 15 min at 60 ℃, immersed in xylene twice for 5 min for deparaffinization, soaked in 100% ethanol (EtOH) twice for 2 min, 95% EtOH twice for 1 min, and 80% EtOH, 70% EtOH, and deionized water for 1 min each for rehydration. Antigen retrieval was performed by boiling the slides for 20 min in antigen unmasking solution (Vector Laboratories, Burlingame, CA, USA). Slides were cooled in buffer for 15 min, rinsed gently with running water for 5 min, and washed with PBS for 5 min. Nonspecific binding was blocked by incubation with CAS block (Life Technologies, CA, USA) for 30 min. Tissue sections were incubated with primary antibodies at 4 °C overnight and washed three times with PBS containing 0.2% Triton X-100 (PBSTx; Sigma-Aldrich, St. Louis, MO). Antibodies against the following were used: CD34 (Abcam, Cambridge, UK), Ki67 (eBioscience, San Diego, CA), beta-catenin (Abcam), LEF1 (Thermo Fisher Scientific, Waltham, MA), c-MYC (Abcam), KRT15 (Abcam), SOX9 (eBioscience), GFP (Invitrogen, Carlsbad, CA), AIMP1 (Thermo Fisher Scientific), and EPRS (Thermo Fisher Scientific). Sections were incubated with secondary antibodies conjugated with Alexa Fluor 488 or 594 (Invitrogen).

After washing three times with PBSTx, 4′, 6-diamidine-2′-phenylindole dihydrochloride (Thermo Fisher Scientific) was added for nuclear counterstaining. Coverslips were mounted onto glass slides with a fluorescent mounting medium (Biomeda Corp., Burlingame, CA). All images were obtained using A1 confocal microscopy (Nikon, Japan).

### Animals

C57BL/6 mice were purchased from Orient Bio (Sungnam, Korea). Offspring were genotyped using PCR-based assays of mouse tail DNA. Animal care was conducted in accordance with the guidance of Seoul National University. All animal experiments were performed following the Guidelines for the Care and Use of Laboratory Animals.

### Western blotting

HFSCs, DPCs, and outer root sheath (ORS) were harvested with lysis buffer (25 mM Tris-HCl, pH 7.5, 100 mM NaCl, 5% glycerol, 0.5% Triton X-100, and 1 mM EDTA) containing a 1× protease inhibitor tablet (Roche, Basel, Switzerland) and a 1× phosphatase inhibitor tablet (Roche). The protein concentration was measured using the Bradford assay. Whole-cell lysates were subjected to gel electrophoresis. Using the semi-dry transfer method, proteins were transferred to polyvinylidene fluoride membranes. The membranes were blocked with 5% skim milk (BD Biosciences, Franklin Lakes, NJ, USA) for 30 min and incubated with each primary antibody diluted in 1% skim milk. Primary antibodies against the following were used: alkaline phosphatase (ALP; Santa Cruz Biotechnology, Dallas, TX, USA), beta-catenin (Cell Signaling Technology, Danvers, MA, USA), N-AIMP1 (Atlas Antibodies, Bromma, Sweden), C-AIMP1 (Novus Bio, Littleton, CO, USA), Akt (Cell Signaling Technology), phospho-Akt (Cell Signaling Technology), ERK (Cell Signaling Technology), phospho-ERK (Cell Signaling Technology), FGFR2 (Abcam, Cambridge, UK), phosphor-FGFR (cell signaling), MMP1 (Thermo Fisher Scientific), beta actin (Santa Cruz Biotechnology) and tubulin (Novus Bio). After washing three times for 5 min each with 1× Tris-buffered saline containing Tween 20 (0.1% TBST), the membranes were incubated for 1 h with horseradish peroxidase (HRP)-conjugated secondary antibodies diluted in 1% skim milk and washed three times for 5 min each with 0.1% TBST. Immunoblot images were acquired after incubation with a western HRP substrate detection solution (Abcam).

### *In vivo* 'patch' hair reconstitution assay

In this study, a 'patch' hair reconstitution assay was performed. To isolate mouse dermal cells, C57BL/6 neonates were euthanized via CO_2_ gas inhalation, and the dorsal skin was excised with a surgical scalpel and incubated overnight with 1 mg/mL collagenase/dispase (Roche). The dermis and epidermis were separated by incubating the skin with 0.25% trypsin/10 mM EDTA in phosphate-buffered saline (PBS) for 15 min at 37 °C. Epidermal and dermal cells were filtered through 70 and 100 µm filters (BD Biosciences), respectively, and centrifuged at 1,500 rpm for 5 min. For three-dimensional (3D) DPC spheroid formation, 2D cultured DPCs were harvested and re-seeded into 96-well HydroCell plates (NUNC, Rochester, NY, USA) at a density of 1 × 10^4^ cells/well. The plates were centrifuged and incubated at 37 °C for 48 h. A total of 100 spheroids were combined with freshly isolated neonatal mouse epidermal cells (1 × 10^6^ cells) and co-implanted subcutaneously into the dorsal skin of 7-week-old female nude mice. After three weeks, the mice were euthanized and the dorsal skin of the mice was excised with a scalpel, and the reconstituted HFs were quantified.

### Statistical analysis

Student's *t*-test was used for statistical analysis. P-values < 0.05 were considered significant.

### Data availability statement

Primary data are available upon request.

## Results

### AIMP1 protects against hair loss by inducing anagen

**Figure 1:**
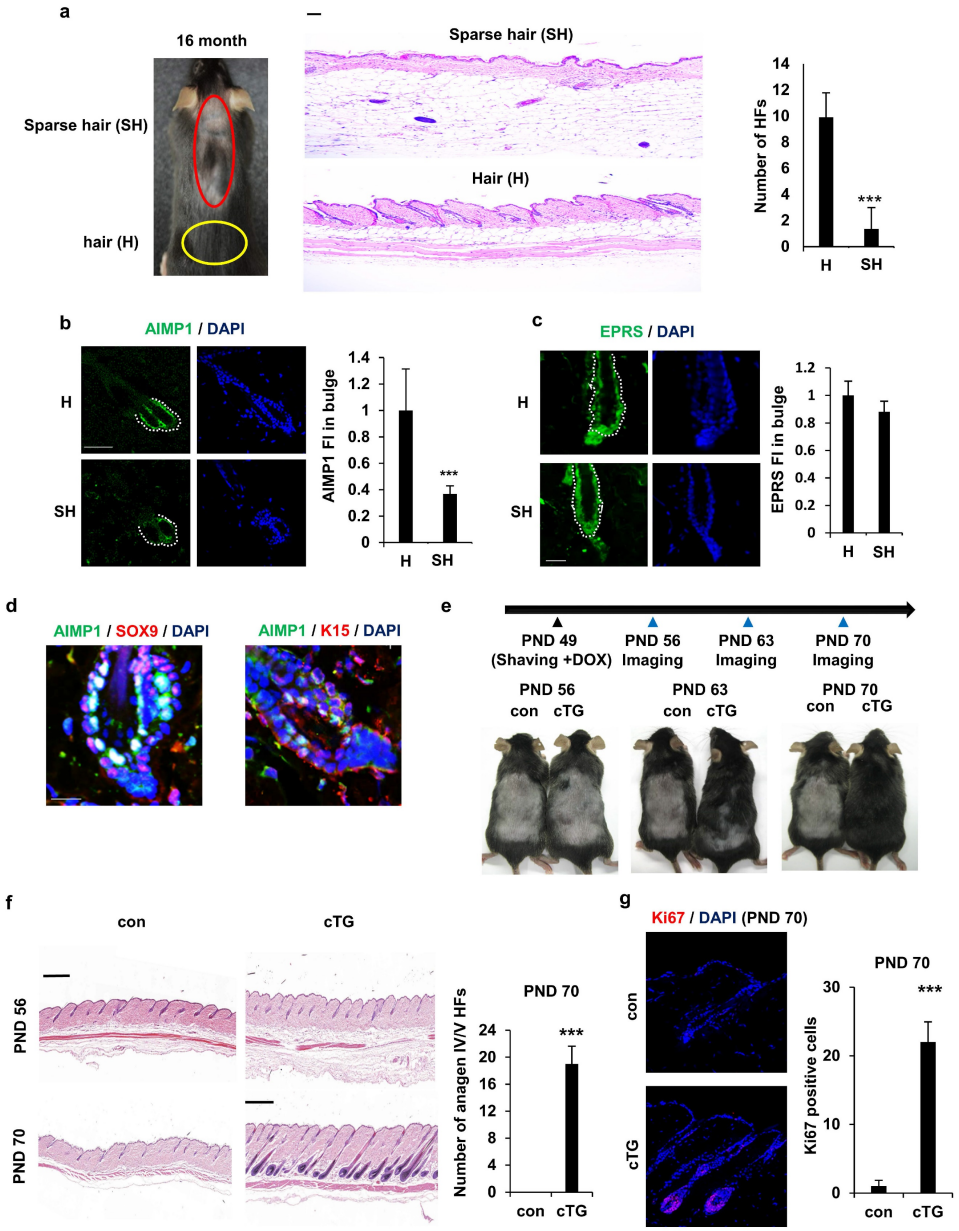
** AIMP1 for hair follicle maintenance**. **(a)** Representative and histological pictures of C57BL/6 (n = 4). HFs counts from each region. **(b)** IF images of AIMP1 in the hair follicle. Relative fluorescence intensity (FI) was measured using Image J. **(c)** IF images of EPRS in the hair follicle from H and SH regions. Bulge was surrounded by dotted line. Relative FI of EPRS in the bulge was measured using Image J software. **(d)** IF images of AIMP1, SOX9, and K15 in the bulge region from 2-month-old C57BL/6. **(e)** The design for analyzing hair growth and representative pictures of mice taken at indicated days (n = 2). **(f)** H&E image from each mouse. HFs counts from each mouse. **(g)** IF staining of Ki67 and counts of Ki67 positive cells from each mouse. The bars: 20 μm (**c**, **d**), 50 μm (**a, b and g**) and 500 μm **(c, f)**. Error bars indicate mean +/- SD. ***: P < 0.001.

To explore the link between AIMP1 expression and HF maintenance, we assessed the correlation between AIMP1 levels and HF count in aged mice. Aged mice exhibited diffuse and symmetric patterns of hair loss on their backs. These patterns typically became obvious in most dorsal areas, where an uneven or linear hair loss pattern formed and spread toward their flanks. Similarly, hair loss in C57BL/6 mice was apparent approximately 16 months after birth. We used 16-month-old mice for histological analysis of HFs in the sparse hair (SH, red circles) and hair (H, yellow circles) regions (Figure 1a). As expected, skin in the SH region exhibited a significant decrease in HFs (Figure 1a). To determine the functional relevance of AIMP1 in HF maintenance, we compared the expression levels of AIMP1 between hair and sparse hair regions using IF staining. AIMP1 was distributed initially evenly in all tissues and was especially enriched in the bulge at the telogen phase (Figure 1b).

AIMP1 levels decreased in the bulges isolated from the SH region (Figure 1b). The fluorescence staining intensity (FI) of AIMP1 was three-fold higher in the H than in the SH regions (Figure 1b). As a negative control, the expression level of glutamyl-prolyl-tRNA synthetase (EPRS), a component of the multi-tRNA synthetase complex, together with AIMP1, was similar between the two regions (Figure 1c) 21. HFSCs reside in the bulge, and AIMP1 is an enriched bulge region. Therefore, we examined the levels of HFSC markers, including SOX9 and K15, along with AIMP1 using IF staining. Interestingly, AIMP1 colocalized with these markers in bulge cells (Figure 1d).

To determine whether overexpression of AIMP1 influences HF maintenance, we generated KRT14cre-AIMP1 conditional transgenic (cTG) mice in which AIMP1 expression was induced in a skin-specific manner (S Figure 1a). The generation of AIMP1 cTG mice was verified using genotyping and IF staining (S Figure 1b, S1c). Mice were clipped on postnatal day (PND) 49 when the second telogen usually takes place, and a picture was taken every week thereafter (Figure 1e) 22. AIMP1 cTG mice entered the anagen phase earlier than wild-type (WT) mice and showed the next hair coat by PND 63 (Figure 1e). Hair was fully regrown by PND 70. This difference in hair regrowth from the dermis to the skin surface was confirmed using hematoxylin and eosin (H&E) staining (Figure 1f). IF staining for the proliferation marker, Ki67 revealed additional Ki67-positive cells in the HFs of AIMP1 cTG mice on PND 70 (Figure 1g).

**Figure 2:**
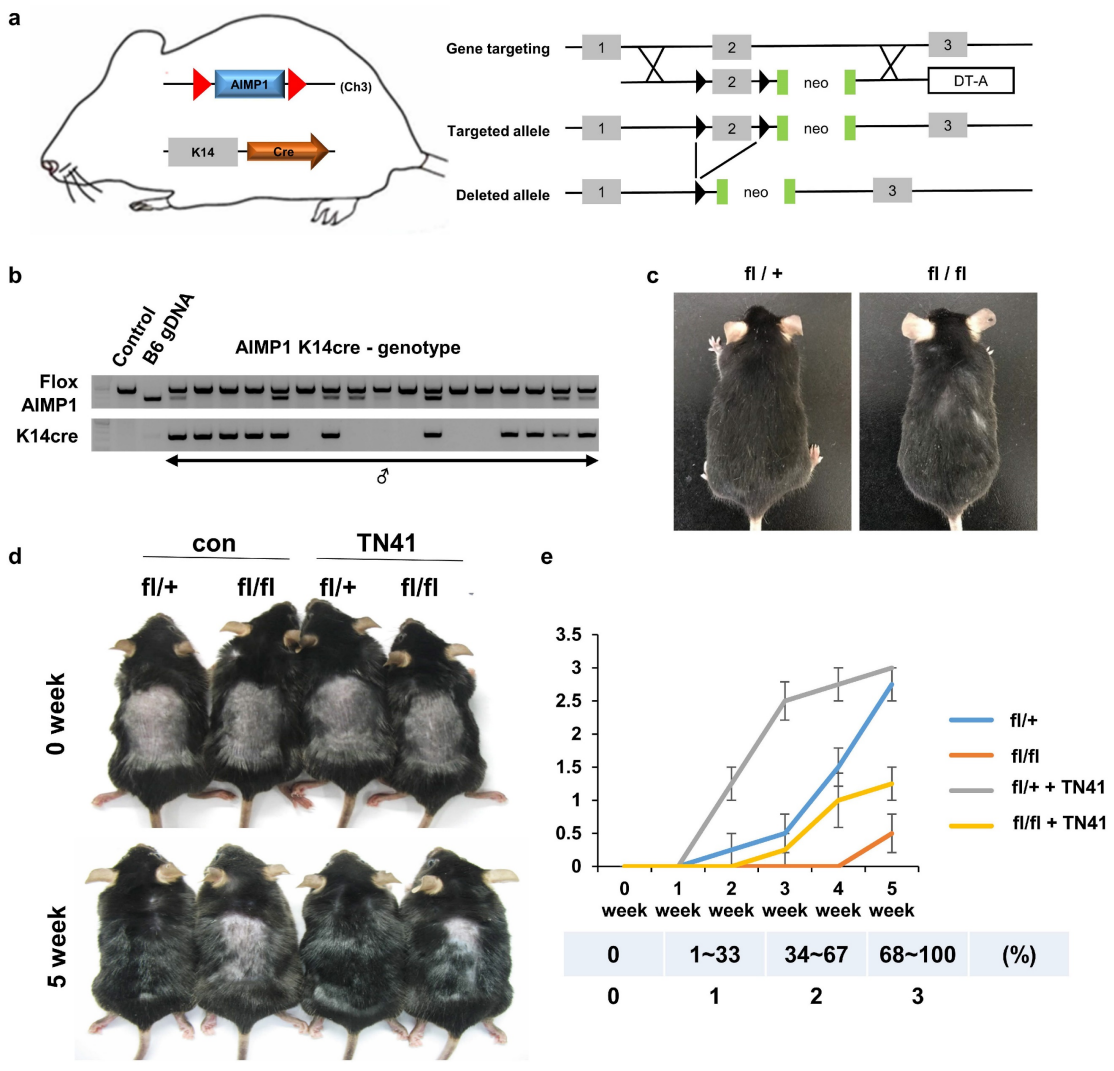
** A. Schematic representation of skin-specific AIMP1 conditional KO mice, AIMP1-KRT14-cre (fl/fl). (a)** Diagram of the mouse AIMP1 locus, targeted allele, and deleted allele. Exon 2 was flanked by the loxP sequence via homologous recombination with the targeting constructs. Flanked exon2 was deleted in the presence of the Cre enzyme. The absence of exon2 disrupts the 1st ATG of the AIMP1 coding sequence. **(b)** Validation of AIMP1 deletion using genotyping. **(c)** Representative KRT14-cre; AIMP1 fl/+ (fl/+) and KRT14-cre; AIMP1 fl/fl (fl/fl) mice at 6 months. **(d)** Representative images of fl/+ and fl/fl mice at 0 and 5 weeks after shaving. Mice were clipped on PND 49 and treated with 100 nM of TN41 or the vehicle (con) once daily (≥2 mice per group). **(e)** Hair growth was measured at 0, 1, 2, 3, 4, and 5 weeks and analyzed using ImageJ software (≥4 mice per group). TN41 (100 nM). Hair growth scoring; 0%: 0, 1-33%: 1, 34-67%: 2, 68-100%. Error bars indicate mean +/- SD. ***: P < 0.001.

Next, we generated systemically inducible AIMP1-transgenic mice (iTG) (S Figure 2a). We confirmed that the mice were correctly generated by analyzing AIMP1 levels using western blot (WB) and IF analysis (S Figure 2b). We clipped the dorsal skin of WT and AIMP1 iTG mice on PND 49 and observed hair cycle progression from telogen to anagen in the two types of mice under native conditions (S Figure 2c).

Four weeks later, AIMP1 iTG mice exhibited hair regrowth on over 80% of their back area, whereas most WT mice showed no hair regrowth (Figure S2d). AIMP1 iTG mice showed more anagen HFs than control mice (S Figure 2e).

Next, we generated HF-specific AIMP1 depletion mice (AIMP1-KRT14-cre) and determined whether AIMP1 depletion affected hair maintenance (Figure 2a). The generated mice were confirmed via genotyping (Figure 2b). At six months old, the homozygous AIMP1-KRT14-cre (fl/fl) mice showed hair loss on the back and neck compared with those of heterozygous AIMP1-KRT14-cre (fl/+) mice (Figure 2c). We hypothesized that deletion of AIMP1 would cause a defect in hair growth; therefore, we examined the effects of AIMP1 depletion on hair regrowth after shaving at PND 49. Whereas the hairs of the fl/+ mice fully recovered at five weeks after shaving, AIMP1 deletion mice showed delayed hair growth (Figures 2d and 2e). After five weeks, the hair regrowth areas of the fl/+ mice were approximately five-fold higher than those of skin-specific AIMP1 knockout mice (Figure 2e). These results suggest the involvement of AIMP1 in the maintenance of HFs and that it induces the transition of the hair cycle from telogen to anagen.

### Mechanism for the cleavage of secreted AIMP1

HF maintenance is primarily regulated by HFSCs and DPCs, with their communication being essential. The crosstalk between these cells regulates the hair cycle 8. AIMP1 acts as an extracellular signaling molecule or an intracellular functional molecule 23, 24, and extracellular AIMP1 possesses stem cell activation functions 19, leading us to hypothesize that extracellular AIMP1 mediates HFSC-DPC communication. We first confirmed whether AIMP1 functioned as an extracellular signaling molecule. AIMP1 was enriched in the bulge, with an expression pattern correlated with HFSCs (Figure 1d).

**Figure 3:**
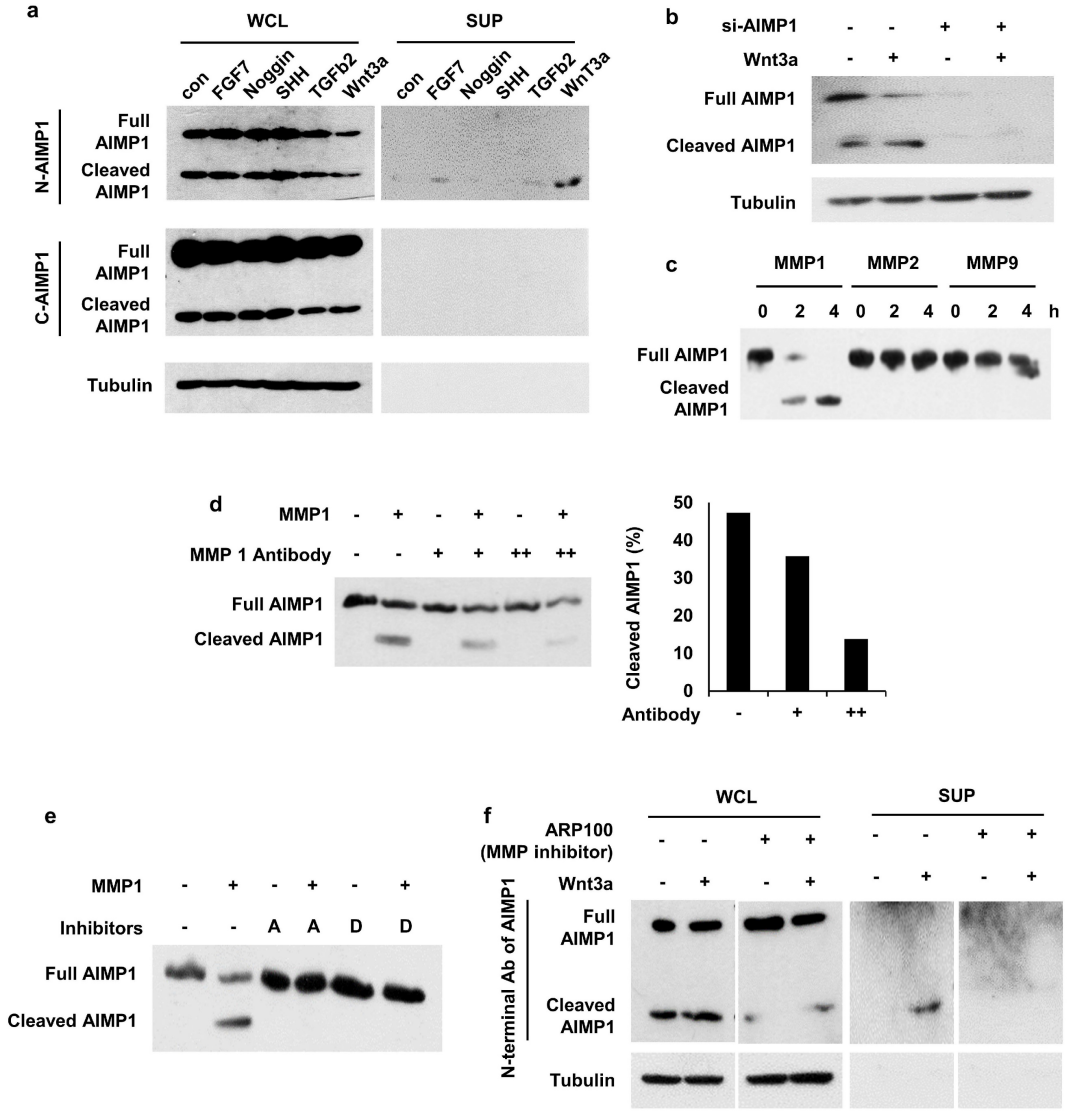
** Secretion of AIMP1 N-terminal fragment from Wnt-activated HFSCs. (a)** AIMP1 secretion by incubating HFSCs under different conditions. The proteins in the whole cell lysates (WCL) and supernatant (SUP) were subjected to WB analysis with the antibodies specific to the N-terminal (N-AIMP1) and C-terminal regions of AIMP1 (C-AIMP1). Wnt3a: 200 ng/ml, FGF7: 100 ng/ml, Noggin: 200 ng/ml, TGFβ2: 10 ng/ml, SHH: 200 ng/ml. **(b)** HFSCs were transfected with AIMP1 siRNA (10 pmol) and treated with Wnt3a (200 ng/ml). WB was performed with the N-AIMP1 antibody. **(c)** AIMP1(400 μg) was incubated with each of MMP1 (72.9 ng), MMP2 (100 ng), and MMP9 (106 ng) at 37 ℃ for 4 h and WB analysis with the N-AIMP1 antibody. **(d)** AIMP1 was incubated with recombinant MMP1 and different amounts of anti-MMP1 antibody. AIMP1 protein was detected by N-AIMP1 Antibody.** (e)** AIMP1 was incubated with MMP1 in the absence or presence of ARP100 (A, 1.5 μg, MMP1 and MMP2 inhibitor) and doxycycline hyclate (D, 1 μg, MMP1, MMP9 and MMP12 inhibitor). **(f)** Secretion of AIMP1 fragment was confirmed by WB in the absence or presence of ARP100.

Therefore, we evaluated the secretion of AIMP1 from HFSCs and its regulation by treating HFSCs with effector molecules. We observed cleaved AIMP1, approximately 23 kDa in size, in the Wnt3a-treated supernatant (Figure 3a) in both cytosol and extracellular space via WB analysis employing an antibody specific to the N-terminal region of AIMP1, but not to its C-terminal region. To determine whether the detected peptide originated from AIMP1, we knocked down AIMP1 using an AIMP1-specific siRNA. The amount of AIMP1 fragment decreased in half following treatment with siRNA against AIMP1 (Figure 3b).

A database-based peptide library was recently used to identify AIMP1 protease 25. AIMP1 is predicted to be a target candidate for matrix metalloproteinase (MMP). We conducted an *in vitro* cleavage assay to confirm whether AIMP1 was a target of MMPs. Among the MMPs, MMP1 cleaved AIMP1 within 2 h (Figure 3c). AIMP1 was completely cleaved after 4 h of incubation with MMP1; the cleaved form of AIMP1 was not detected when MMP1 was blocked with inhibitors or a high concentration of MMP1 antibody (Figure 3d, 3e). We performed a secretion assay with inhibitors of MMP1 to determine whether MMP1 influences the secretion of AIMP1 N-terminal peptides from cells. The secreted fragment of AIMP1 disappeared when the ARP100, an MMP inhibitor, was co-treated with Wnt3a (Figure 3f). MMP1 is an extracellular protease. However, the intracellular activity of MMP1 has recently been reported. In particular, skin photo-aging is associated with the acceleration of collagen degradation by the intracellular activity of MMP1 26-28. The results indicate that AIMP1 exists in its full-length and MMP1-truncated forms in HFSCs, with the truncated form being secreted in response to Wnt3a signaling.

### Determination of the active domain of AIMP1 responsible for hair-growing activity

**Figure 4:**
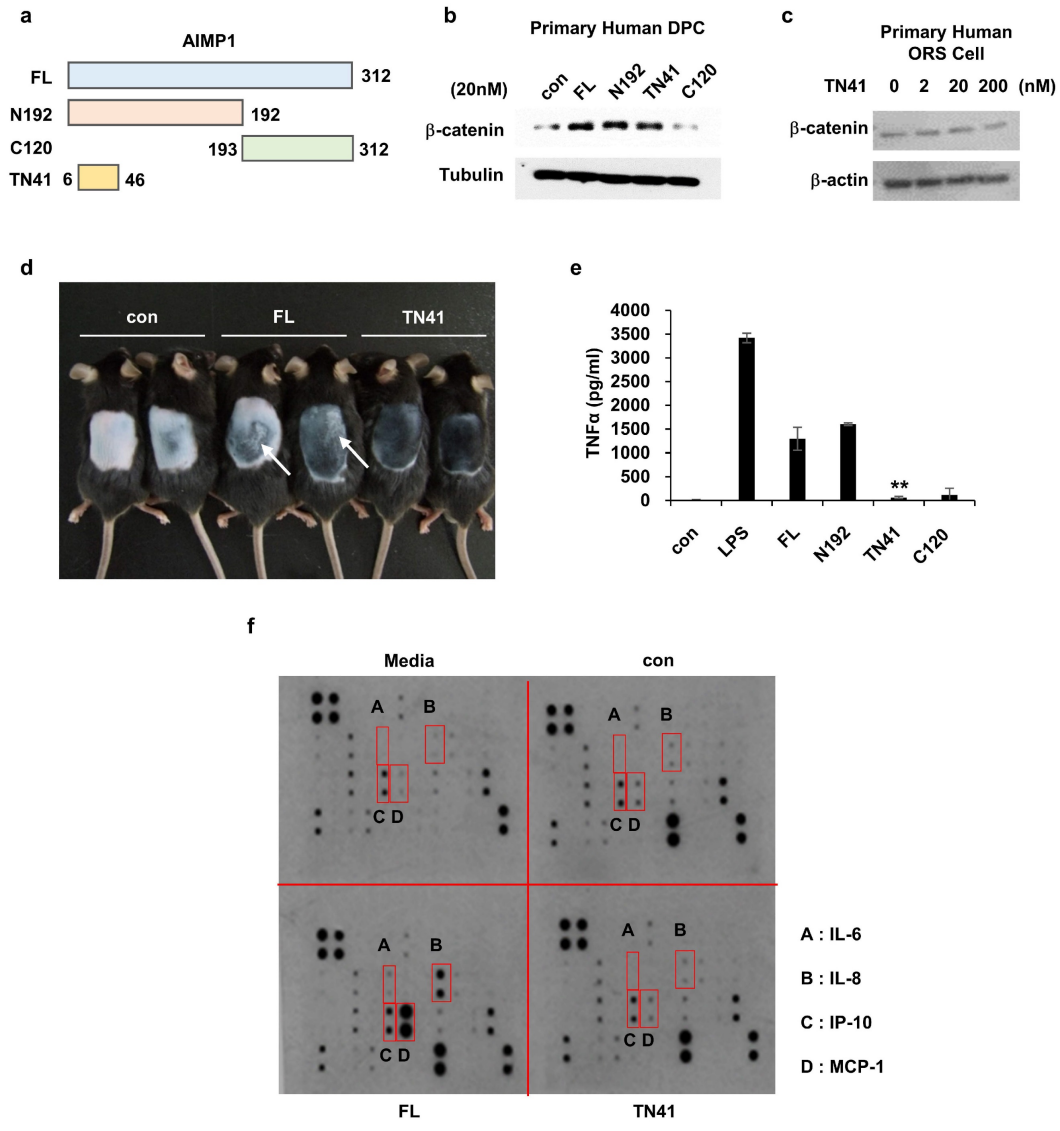
** Determination of the peptide region of AIMP1 responsible for the hair growing activity without inflammation. (a)** Diagram of fragments of AIMP1. FL: Full length, N192: 1-192aa, C120: 193-312aa, TN41: 6-46aa. **(b-c)** β-catenin induction was tested by human DPC and ORS cells with AIMP1 full length and fragments. Human primary DPCs were treated with FL, N192, TN41, or C120 (20 nM each). Human primary ORS cell was treated with each concentration of TN41. WB were performed with each antibody. **(d)** Mouse back skins were depilated at PND 49, and images were taken PD10. White arrows indicate the skin regions showing inflammatory response. **(e)** Effects of AIMP1 fragments on TNF-α secretion from RAW 264.7 cells. **(f)** Effects of FL and TN41 on cytokines secretion from DPC. Media: culture medium, con: cultured medium containing vehicle. The cytokines secreted by FL but not by TN41 are in red boxes.

Figure 3 demonstrates that the truncated form localizes to the N-terminal region of AIMP1. The domain of AIMP1 responsible for promoting hair growth was characterized to corroborate this finding. To characterize the functional domain of secreted AIMP1 involved in hair growth, we synthesized four AIMP1 peptides based on a previous study (Figure 4a) 18 and used them to treat DPCs and outer root sheath (ORS) keratinocytes to identify the region active in hair regrowth. We analyzed the accumulation of β-catenin in both cell types as it is a critical molecule in hair growth and maintenance in the HF microenvironment 29. Full-length AIMP1 (FL), N192, and TN41, but not C120, elevated β-catenin levels in human DPCs (Figure 4b). By contrast, TN41 did not increase β-catenin levels in the ORS cells (Figure 4c).

Inflammatory responses have been linked with hair cycle disruption and the onset of alopecia 30, 31. Notably, FL, but not TN41, elicited inflammatory responses, such as skin swelling, consistent with its known pro-inflammatory activity (Figure 4d). To validate this effect, RAW 264.7 mouse macrophages were treated with the four AIMP1 peptides, and TNF-α secretion, a hallmark cytokine of inflammation, was assessed. Both FL and N192, such as lipopolysaccharide (LPS), induced TNF-α secretion, whereas TN41 and C120 did not (Figure 4e). Next, we verified the presence of inflammatory cytokines in DPCs following treatment with FL and TN41. FL, but not TN41, induced the secretion of inflammatory cytokines, including interleukin (IL)-6), IL-8, monocyte chemoattractant protein-1 (MCP-1), and interferon gamma-induced protein 10 (IP-10) (Figure 4f). This analysis confirmed that the AIMP1 N-terminal fragment secreted by HFSCs lacks pro-inflammatory activity that can negatively impact hair growth; instead, it acts on DPCs to promote hair growth and maintenance.

### Effect of AIMP1 peptide treatment on hair growth

**Figure 5:**
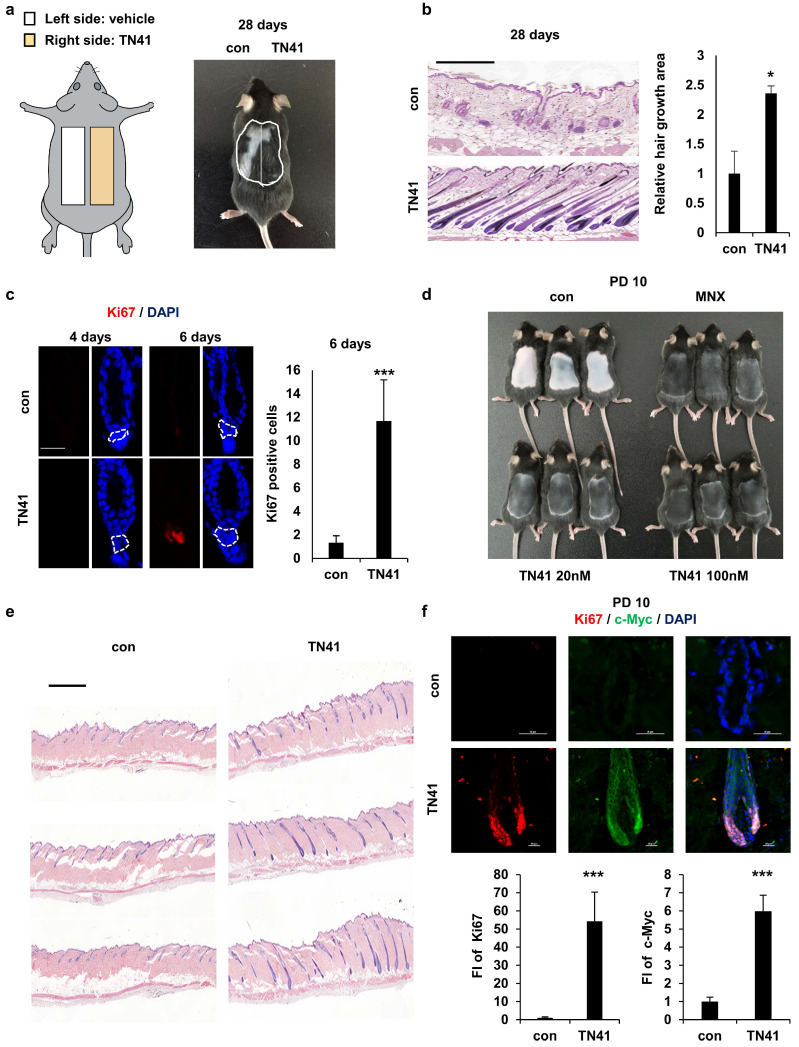
** Effect of an AIMP1-derived peptide on hair growth. (a)** Mouse back skins were clipped at PND 49. The left and right halves of the back skin were treated with vehicle, TN41 (100 nM), respectively. **(b)** Representative H&E image at 28 days after clipping (n = 3). HFs area was measured. **(c)** IF images of Ki67 and counts of Ki67- positive cells in hair follicles. **(d)** Mice were depilated on PND 49 and treated with the vehicle, 3% MNX and TN41. Pictures were taken on PD10 (n = 3). **(e)** H&E image of control and the TN41 (100 nM) treated dorsal skin.** (f)** Representative IF staining images for c-Myc and Ki67 in hair follicles and measured relative FI. The bars: 200 μm **(b)**, 20 μm **(c, d and f),** 500 μm **(e).** Error bars: mean ± SD. ***: P < 0.001.

To evaluate the efficacy of TN41 in promoting hair growth via HFSC-DPC interactions, it was examined in an animal model via topical application. First, we monitored how TN41 reached HFs using TN41 conjugated with a red fluorescent dye and determined that TN41 flowed through the HF pores (S Figure 3a). We previously observed that AIMP1 inducible mice entered the anagen phase earlier, such that their hair regrowth rates differed from those of WT mice. To determine the telogen-to-anagen transition effect of TN41 on hair growth, we clipped the dorsal hair of C57BL/6 mice and applied vehicle and TN41 on the left and right sides of the clipped region, respectively (Figure 5a). Hair growth was verified using histological analysis. After treatment with TN41 for four weeks, the TN41-treated regions exhibited more hair regrowth than the vehicle-treated regions (Figure 5b). Additionally, we performed IF staining to identify the expression of Ki67 in HFs. A higher staining intensity of Ki67 was observed in the secondary hair germ of TN41-treated mice six days after clipping (Figure 5c), and the Ki67 signal was observed in the matrix cells (S Figure 3b).

To assess the impact of TN41 on hair growth rates, mice were depilated to synchronize the hair cycle at the onset of the anagen phase. The depilated mice were treated with TN41 and minoxidil (MNX), a hair growth-promoting agent, as the positive control. Hair was observed on the dorsal skin of mice in both the TN41-treated and MNX-treated groups, whereas hair growth was not apparent on the skin of vehicle-treated mice at 10 days post-depilation (PD10) (Figure 5d). Hair growth was also verified using histological analysis (Figure 5e). We confirmed this result by examining Ki67 and c-Myc levels in HFs using IF staining. Higher fluorescence staining of Ki67 and c-Myc was observed in TN41-treated HFs, as they exhibited faster growth than vehicle-treated HFs (Figure 5f).

In the case of AIMP1-KRT14-cre mice, the TN41-treated group showed earlier hair growth initiation and a twofold faster hair growth rate than the untreated group in both fl/+ and fl/fl mice (Figure 2d and 2e). These findings demonstrate that the topical application of TN41 modulates hair growth.

### Mechanism of AIMP1 peptide in hair growth

**Figure 6:**
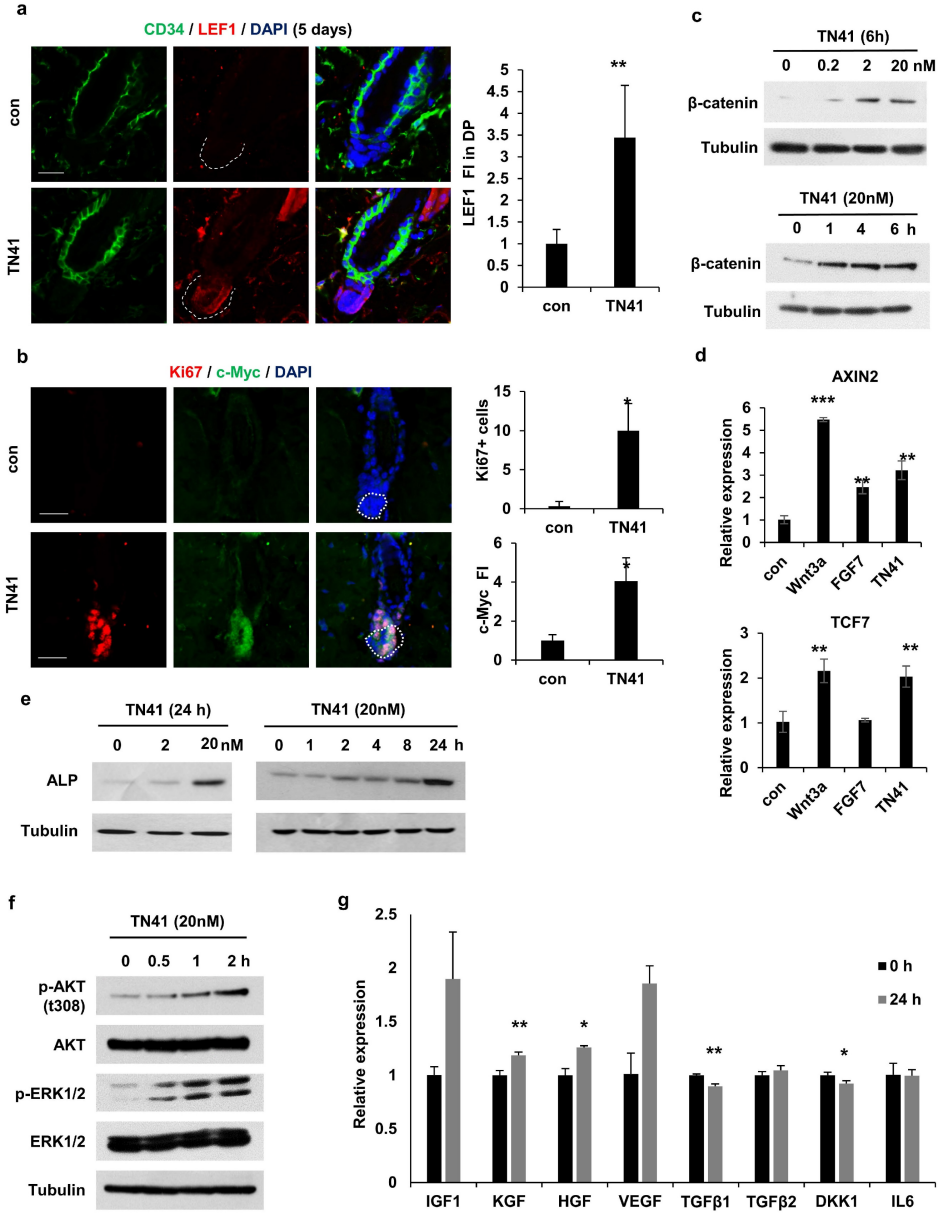
** TN41 induced Activation of the MAPK pathway in DPCs. (a)** IF images of CD34 (HFSC marker) and LEF1 in hair follicles from TN41-treated and vehicle-treated mice. Relative LEF1 FI was measured in the DPC. **(b)** IF images of Ki67 and c-Myc in the hair follicle. Ki67-positive cell was counted and the relative FI of c-Myc was measured in the DPC. **(c and e, f)** DPCs were treated with time and dose-dependent TN41, and WB were performed via each antibody. **(d)** DPCs were treated with 5.1 nM of Wnt3a, 8.9 nM of FGF7, and 20 nM of TN41 for 14 h, and relative gene expression levels were measured by quantitative PCR. **(g)** Regulatory effect of TN41 on the expression of various growth factors in DPCs. The bars: 20 μm (**a** and **b**). Error bars: mean ±SD. ***: P < 0.001, **: P < 0.01, *: P < 0.05.

Together with the β-catenin accumulation in DPCs via TN41 (Figure 4b), to identify the target cells of truncated AIMP1 *in vivo*, we used TN41 to treat the mouse dorsal skin and examined the expression level of lymphoid enhancer-binding factor 1 (LEF1), a transcription factor of Wnt/*β*-*catenin* pathway, in DPCs. TN41-treated mice exhibited high LEF1 expression (Figure 6a). Ki67 and c-Myc were also highly expressed in DPCs following treatment with TN41 (Figure 6b). Additionally, β-catenin levels in DPCs were increased by TN41 in dose- and time-dependent manners (Figure 6c). The levels of *AXIN2* and *TCF7*, regulated by β-catenin, were also increased in DPCs via TN41 (Figure 6d). The expression of alkaline phosphatase (ALP), an established dermal papilla marker related to hair inductivity, was increased following treatment with TN41 in dose- and time-dependent manners (Figure 6e). The N-terminal region of AIMP1 induces β-catenin expression in human bone marrow-derived MSCs via activation of Akt and ERK 19. We determined whether TN41 functions via a mechanism such as that in MSCs of DPCs. Akt phosphorylation at residue 308 increased in a time-dependent manner following treatment with TN41 (Figure 6f). ERK phosphorylation revealed a similar pattern to that of Akt.

Next, we evaluated the gene expression levels of secretory molecules in the DPCs. The expression levels of four positive genes involved in hair growth, namely *KGF*,* HGF*,* IGF*, and *VEGF*, were increased by TN41 (Figure 6g), whereas the expression levels of *TGFb1*,* TGFb2*,* DKK1*, and *IL6*, inhibitory genes for hair growth, were not. The expression of *TGFb1* and *DKK1* was reduced. Therefore, TN41 activates DPCs in *in vivo* and* in vitro* models, leading to the secretion of hair growth-related molecules for intercellular communication. The AIMP1 fragment secreted by HFSCs is postulated to activate DPCs, thereby inducing the secretion of growth factors and enhancing hair growth.

### Effect of TN41 on human hair shaft elongation in cultured HFs

**Figure 7:**
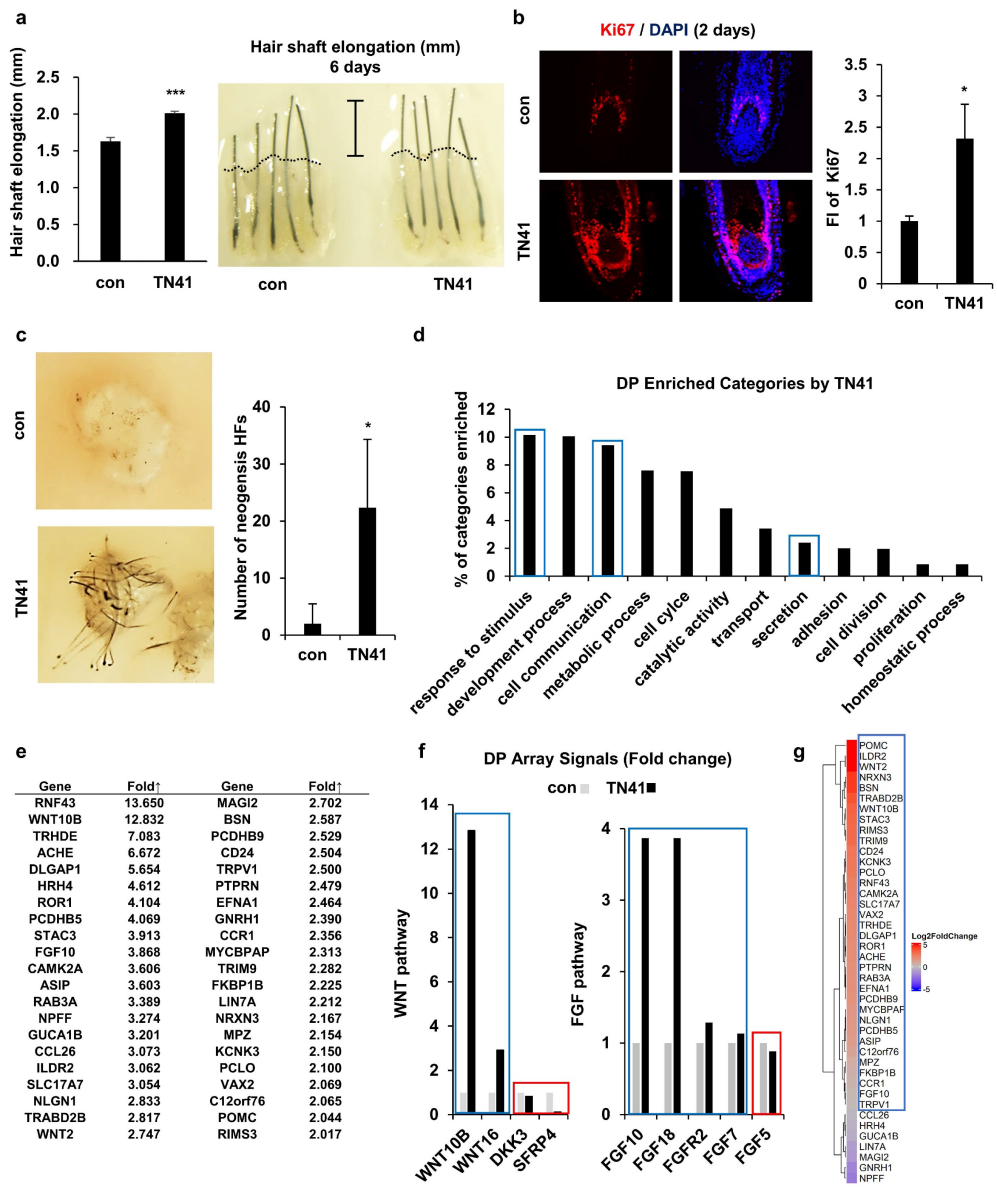
**Effects of TN41 on human DPCs. (a)** Effects of TN41 in human hair shaft elongation. Human HFs were cultured for 6 days with TN41, or the vehicle and hair length were measured (3 donors). **(b)** IF image of Ki67 in human hair follicles and relative FI was measured by Image J. **(c)** 3D-cultured DPCs with or without TN41 were injected into the hypodermis of nude mice. Images were obtained after 2 weeks (injection site = 3) and measured neogenesis HF numbers. **(d)** Gene categories enriched in TN41-treated DPCs measured by RNA sequence. Extracellular signaling molecules were among the most enriched (blue box). **(e)** List of upregulated DPC genes in the extracellular signaling category. **(f)** TN41 treatment induced Wnt and FGF pathway genes in DPC. The microarray signal values of Wnt and FGF pathway members displayed temporal differences. **(g)** RNA-seq heatmap depiction of 42 DPC genes upregulated by TN41 in DPCs co-cultured with HFSCs and treated with vesicles and Wnt3a (shown in Fig. 7e). The fold changes (FC) were shown by color gradient. Blue box: positive genes, red box: negative genes. Error bars: mean ± SD. ***: P < 0.001, *: P < 0.05.

We conducted a hair shaft elongation assay to determine whether AIMP1 had similar activity in human HFs. Since it was not possible to observe the promotion of telogen-to-anagen activation in cultured human HFs, we showed that TN41 could affect human HFs by activating DPCs. Human HFs were cultured in the presence or absence of TN41. After six days, we measured the length of HFs. TN41-treated human HFs showed approximately 30% greater elongation than untreated controls (Figure 7a). Additionally, the number of Ki67-positive matrix keratinocytes around the DPCs was increased in TN41-treated HFs compared with untreated HFs (Figure 7b).

Next, we performed a patch hair reconstitution assay to determine whether TN41 could improve the hair-inductive activity of DPCs. 3D cultured human DPCs (100 spheres; 10^6^ cells) were treated with TN41 for 4 h and co-transplanted with mouse epidermal cells (10^6^ cells) into the dorsal skin of nude mice. Hair induction increased after transplantation of TN41-treated human DPC spheres compared with untreated spheres (Figure 7c).

### TN41-mediated gene expression regulation in human DPCs

We compared the gene expression profiles of untreated and TN41-treated DPCs using microarray analysis and observed numerous differentially expressed genes between these groups. We derived the molecular signatures of genes with greater than two-fold upregulation or downregulation in TN41-treated DPCs relative to their untreated controls and performed bioinformatics analysis to classify these genes. The transcription of DPCs dynamically changed following treatment with TN41 (Figure 7d). Notably, the transcription of genes encoding putative hair cycle stimulating factors within the “extracellular signal” category was enhanced, and further analyses revealed elevated expression of 42 dermal papilla genes in the signaling category (Figure 7e). Notably, Wnt10b expression was highly upregulated by TN41. Wnt10b promotes HF growth and regeneration via the canonical Wnt signaling pathway 32. Next, we focused on the Wnt signaling pathway since Wnt2 and Wnt10b were upregulated by TN41 (Figure 7e). Wnt16 activates human keratinocyte proliferation and differentiation via the non-canonical Wnt transduction pathway 33. DKK3 and SFRP4 are potent inhibitors of the Wnt signaling pathway 34, 35. *FGF10* and its relative gene *FGF18* were identified as DPC signature genes elevated by TN41 (Figure 7e). Consistent with previous findings 36-38, our results suggest a paracrine role for FGF10 and/or FGF18 signaling in epidermal wound repair and hair growth (Figure 7f). To confirm the importance of secreted AIMP1 for the crosstalk between HFSCs and DPCs, we conducted the co-culture system of the two cells separated by a Transwell chamber. Among the TN41-induced 42 genes in DPCs shown above, the expression of 25 genes was increased by more than 1.5-fold, and 35 genes were increased, further supporting the role of AIMP1 or TN41 for the communication between the two cells (Figure 7g, S4). These findings strongly suggest that AIMP1 fragments secreted by HFSCs activate DPCs and validate the efficacy of TN41 in stimulating human hair growth. This underscores the potential for TN41 in clinical applications.

## Discussion

Recent studies have revealed the complexity of cellular and molecular regulators within the skin stem cell niche during development, homeostasis, injury, and aging. Although many putative niche factors have been identified, crosstalk between HFSCs and DPCs remains unclear. In this study, we identified the function of AIMP1 in the HF environment. Wnt-activated HFSCs secrete truncated N-terminal AIMP1, which acts in DPCs, activating the MAPK pathway; this, in turn, leads to the accumulation of β-catenin in DPCs. These processes may trigger the secretion of anagen-inducing molecules from DPCs. Our results are consistent with data showing that AIMP1 enhances the proliferation of bone marrow-derived MSCs via the accumulation of β-catenin 19. Homeostatic signals governing the connections between HFSCs and the surrounding cells remain unclear 39, 40; therefore, additional studies on AIMP1 remain warranted.

Communication between HFSCs and DPCs leads to physical and structural development, maintenance, and HF cycling. Soluble factors derived from these two cells contribute to microenvironmental changes during the asynchronous cycle. Ensuring the balance between apoptosis-associated factors, inducing the anagen-to-catagen transition via apoptosis and growth-associated factors, and maintaining the anagen phase through growth promotion is essential. Alopecia-inducing factors, including hormones, inflammatory cytokines, and senescence molecules, disrupt communication between HFSCs and DPCs by inducing apoptosis or senescence of HFSCs and DPCs. Our study identified a critical role for AIMP1 in mediating the crosstalk between HFSCs and DPCs. To our knowledge, this is the first study that provides evidence that AIMP1 is secreted by HFSCs and regulates DPC function using a co-culture system. Further studies that confirm AIMP1 secretion and its regulatory function on DPCs in alopecia can establish AIMP1 as a promising therapeutic target for hair loss treatment.

Although growth-related factors are potential drug targets, AIMP1 is dual functional protein, making it challenging to use as a therapeutic agent. Several studies, including this one, have demonstrated that AIMP1 activates both inflammation and stem cells, as well as DPCs. Therefore, to utilize AIMP1 as a drug for hair growth, its DPC activation region must be identified. In this study, we identified TN41, a region of AIMP1 that activates DPCs without inducing an inflammatory response, that exhibited a more stable structure than the full AIMP1 protein and, due to its small size, is amenable to peptide synthesis 41. These characteristics suggest TN41 or smaller peptides derived from TN41 as promising candidates for treating hair loss.

Moreover, AIMP1 presents a potential therapeutic target for age-related hair loss. In this study, we confirmed that AIMP1 levels decreased with aging. Additionally, we demonstrated that this reduction impairs the timely progression of the telogen-anagen transition, whereas TN41 enhances this transition. In other words, diminished AIMP1, which affects HFSC-DPC communication, may result in insufficient DPC activation and a disrupted telogen-anagen cycle. Therefore, restoring AIMP1 levels could provide a viable treatment strategy. However, the precise mechanisms underlying the age-related decline of AIMP1 remain to be elucidated. Future research aimed at identifying the transcriptional regulators of AIMP1 and elucidating its aging-associated regulatory mechanisms, along with the discovery of compounds capable of reversing this decline, will facilitate the development of promising therapeutics for hair loss.

Our results demonstrate that AIMP1 participates in the maintenance of HFs, especially by promoting the hair cycle transition from the telogen to anagen phases. This peptide may mitigate hair loss since TN41 enhances hair growth rate and telogen-to-anagen transition in a mouse model and hair shaft elongation in cultured human HFs.

Moreover, the enhanced hair-inducing activity of cultured human DPC spheroids following TN41 treatment suggests that AIMP1 is useful as an adjuvant in cell-based therapy to overcome hair loss.

## Supplementary Material

Supplementary materials and methods, figures.

## Figures and Tables

**Figure 8:**
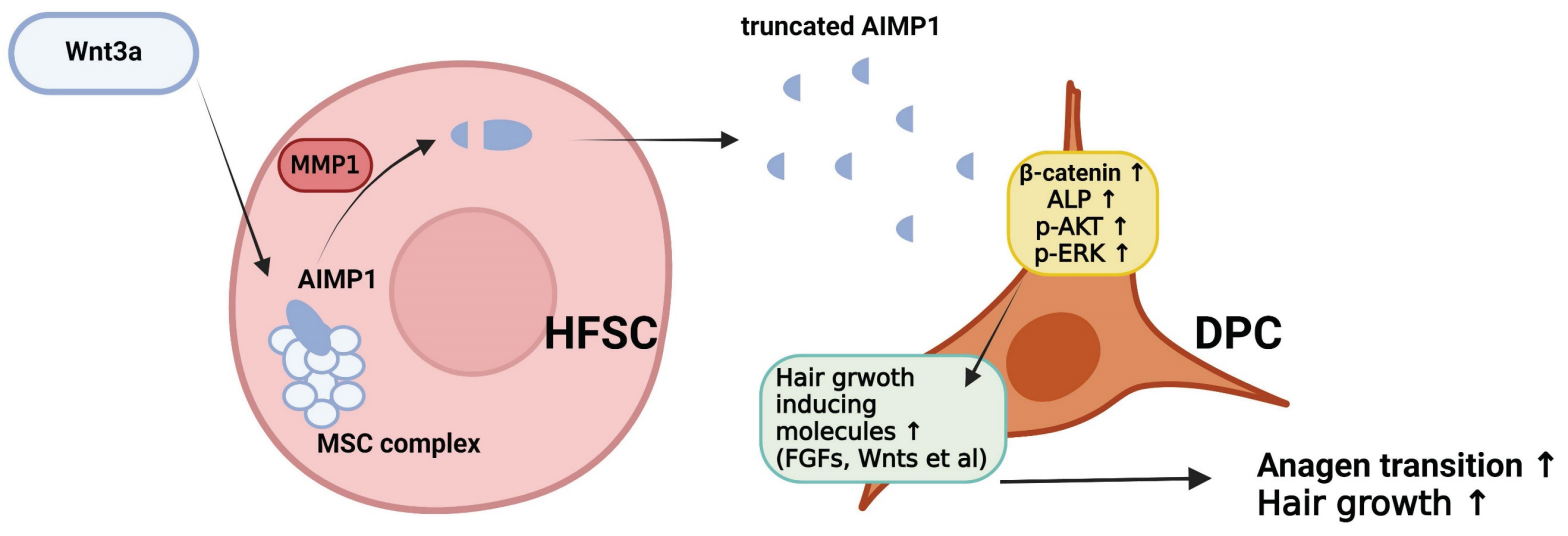
** The schematic diagram illustrating how truncated AIMP1 stimulates DPCs to promote hair growth.** Wnt3a stimulation induces AIMP1 cleavage via MMP1 activation in hair follicle stem cells (HFSCs), leading to the release of a truncated form of AIMP1. The secreted AIMP1 enhances hair growth by upregulating hair growth-promoting molecules in dermal papilla cells (DPCs), subsequently activating β-catenin, ALP, p-AKT, and p-ERK signaling pathways, thereby facilitating anagen transition and promoting hair growth.

## Abbreviations

HFSCs: hair follicle stem cell
DPCs: dermal papilla cells
ORS: outer root sheath
AIMP1: aminoacyl-tRNA synthetase-interacting multifunctional protein1
HFs: hair follicles
FI: fluorescence staining intensity
H&E: hematoxylin and eosin
PND: postnatal day IF: immunofluorescence
WB: western blot
cTG: conditional transgenic
PND: postnatal day
PD: post-depilation
