# Supplementary material for: AIMP1-Derived Peptide Secreted from Hair Follicle Stem Cells Promotes Hair Growth by Activating Dermal Papilla Cells
Source: Int J Biol Sci. 2024 Oct 21;20(14):5764–78. doi: 10.7150/ijbs.101127 (PMC11528461; doi:10.7150/ijbs.101127)

Materials and methods

In vitro cleavage assay

AIMP1 and MMP1,2 and 9 (Novus Bio) were incubated in cleavage assay buffer (20 mM HEPES pH 7.4, 140 mM NaCl, 2 mM CaCl2) for 4 h at 37℃. ARP100 (Santa Cruz Biotechnology), doxycycline hyclate (Sigma-Aldrich, St. Louis, MO, USA) and anti-MMP1 antibody (R&D Systems, Minneapolis, MN, USA) were used for each assay. Proteins were separated by sodium dodecyl sulfate polyacrylamide gel electrophoresis (SDS-PAGE) and subjected to immunoblotting.

Hair depilation and peptide administration

For hair cycle induction, hair on the dorsal skin of 7-week-old mice was depilated to induce a new hair cycle after confirming that the skin had a light pink color, which indicates that the HFs are synchronized at the telogen phase. For peptide treatment, 150 μL of peptides in carbomer (20 and 100 nM) were topically applied to the depilated area once per a day. We used thioglycolate hair removal cream (Ildong Pharmaceutical, Seoul, Korea) for chemical depilation.

Isolation and culture of human HFs

The Medical Ethical Committee of Kyungpook National University (Daegu, Korea) approved all described experiments (IRB No. KNU-2021-0113). Occipital scalp biopsy specimens were obtained during hair transplantation in human male patients with androgenetic alopecia. The Medical Ethical Committee of Kyungpook National University Hospital (Daegu, Korea) approved all described experiments. HFs were isolated from non-balding scalps as previously described with minor modifications [21, 22]. Briefly, the subcutaneous fat portion of the scalp

skin, including the lower HFs, was dissected from the epidermis and dermis. HFs were then isolated under a binocular microscope using forceps. Isolated HFs were maintained in Williams E media (Sigma-Aldrich) supplemented with 2 mM L-glutamine, 100 U/mL streptomycin, and 10 ng/mL hydrocortisone. Follicles were maintained in a humidified atmosphere of 5% CO_2 at 37 °C.

Cell culture

The dermal papilla was isolated from the bulbs of dissected HFs, transferred onto plastic dishes coated with bovine type I collagen, and cultured in DMEM supplemented with 1% antibiotic-antimycotic solution and 20% heat-inactivated FBS at 37°C in a humidified atmosphere containing 5% CO_2. These explants were incubated for several days, and the medium was changed every three days. Once sub-confluent, the cells were harvested with 0.25% trypsin/10 mM EDTA in PBS, split at a 1:3 ratios, and maintained in DMEM supplemented with 10% FBS and 1ng/mL of FGF2. Non-balding scalp specimens were obtained from patients undergoing hair transplantation surgery. The ORS cells isolated from HFs were cultured as described before [23]. Briefly, the hair shaft and hair bulb regions of the HF were cut off to prevent contamination with other cells. Trimmed HFs were immersed in DMEM supplemented with 20% fetal bovine serum. On the third day of culture, the medium was changed to keratinocyte growth medium (Gibco BRL) containing penicillin, streptomycin, and fungizone. After subculture, cells were maintained in keratinocyte growth medium and cells from the second passage were used in this study. HFSCs were purchased from Cellprogen (Torrance, CA, USA). HFSCs were used before passage six.

Reverse transcription PCR and real-time PCR

Total RNA was isolated from DPCs by using the RNeasy Mini Kit (Qiagen, Hilden, Germany), according to the manufacturer's protocol. Complementary cDNA was synthesized by RevertAid First Strand cDNA Synthesis Kit (Thermo Fisher Scientific), according to the manufacturer's protocol. The real-time PCR was performed using Step one Plus real time PCR Assay (Applied Biosystems, Foster City, CA). All reactions were carried out using Power SYBR Green premix (Applied Biosystems).

TNF-α ELISA assay

RAW264.7 mouse macrophages (2 × 104) were cultured in 24-well plates containing DMEM with 10% FBS and 1% antibiotics for 12 h and then treated with the purified protein for 6 h. The media were harvested after centrifugation at 3,000 ×g for 5 min, and secreted TNF-α was measured using a TNF-α ELISA kit (BD Biosciences) following the manufacturer's instructions.

Microarray analysis

Total RNA was extracted with the RNeasy Micro Kit. The Illumina NextSeq500 array (San Diego, CA, USA) of EBiogen (Seoul, Korea) was used. To analyze the Gene Ontology (GO) terms with ≥2.0-fold increased or decreased gene clusters, the web-based Database for Annotation, Visualization and Integrated Discovery (DAVID) 6.7 was used. To extract reliable GO terms belonging to biological processes, Fisher's exact test and multiple test correction ($p < 0.05$) were used.

Determination of AIMP1 secretion

HFSCs were cultivated to 60% confluence in DMEM containing 10% FBS. Cells were washed twice and transferred to serum-free DMEM for 2 h. Cells were treated with Wnt3a (R&D Systems), Noggin (Miltenyi Biotec), FGF7 (Sigma-Aldrich), sonic hedgehog (SHH) (Miltenyi Biotec), or transforming growth factor (TGF)-β2 (Thermo Fisher Scientific) followed by incubation for 24 h. The culture media were collected and centrifuged at 500 ×g for 10 min. Supernatants were centrifuged again at 10,000 ×g for 30 min to eliminate membrane organelles, and the proteins were precipitated with 12% trichloroacetic acid and incubated for 12 h at 4°C. Pellets were obtained by centrifugation at 18,000 ×g for 15 min and neutralized with 100 mM HEPES, pH 8.0. Protein in the pellets were separated by SDS-PAGE and subjected to immunoblotting.

Preparation of paraffin and frozen sections

For paraffin sections, mouse skin or human HF was fixed in 10% formalin solution at 4°C overnight and then embedded in paraffin. Paraffin-embedded specimens were cut into 5 µm-thick sections. For frozen sections, mouse skin or human HF tissues were immersed in ice cold 4% paraformaldehyde in PBS (pH 7.4) for 10 min. The fixed samples were embedded in optimal cutting temperature compound (Sakura FineTechnical Co., Ltd), snap-frozen in liquid nitrogen, and stored at –80°C. Frozen samples were cut in 10 µm-thick sections.

Hematoxylin and eosin staining

Paraffin sections were deparaffinized and then rehydrated. Frozen sections were removed from the optimal cutting temperature compound and stained with hematoxylin and eosin (H&E; Sakura FineTechnical Co., Ltd., Tokyo, Japan) for tissue histology analysis using an optical microscope.

Cytokine array

DPCs were cultured in 12-well plates containing DMEM with 10% FBS, 1 ng/mL of FGF2, and 1% antibiotics for 24 h and then treated with the AIMP1 FL (20 nM) and TN41 (20 nM) for 12 h. Effect of AIMP1 FL and TN41 on the secretion of different cytokines was determined using cytokine array (R&D Systems) following the manufacturer's instructions.

Co-cultivation of HFSC and DP cells in transwell chamber

DP cells were seeded at a density of 1×10^6 in the upper chamber of the Trans well (Corning, REF: 3419, 100mm dish/0.4μm: 12/Cs), and cultured in Dulbecco's modified Eagle's medium (DMEM; HyCloneTM, Utah 84321, USA) supplemented with 10% heat-inactivated fetal bovine serum (FBS, Australian origin, HyCloneTM), 1ng/ml bFGF, and 1% antibiotic-antimycotic solution (Gibco BRL, Gaithersburg, MD, USA). Human Hair Follicle Stem Cells (HFSC, Celprogen Inc., CA, USA) were seeded at a density of 1×10^6 in the lower chamber after coating with Matrigel hESC-qualified Matrix (Corning) and cultured in Human Hair Follicle Stem Cell Culture Complete Expansion Media with serum and antibiotics (Celprogen). After separately culturing the upper and lower chambers for 24 hours, the medium for the upper DP cells was replaced with Human Hair Follicle Stem Cell Culture Complete Expansion Media serum-free with antibiotics for 12 hours. For the lower HFSCs, 250 ng/ml Wnt3a was added to the same medium as the DP cells at the same times. Before co-culturing the prepared cells from the upper and lower chambers, they were divided into groups that were washed with PBS and groups that were not washed. After 48 hours of co-culture, DP cells were collected and storage at -70°C for RNA seq analysis.

RNA sequencing analysis

Total RNA concentration was calculated by Quant-IT RiboGreen (Invitrogen, #R11490). To assess the integrity of the total RNA, samples are run on the TapeStation RNA screentape (Agilent, #5067-5576). Only high-quality RNA preparations, with RIN greater than 7.0, were used for RNA library construction.

A library was independently prepared with 0.5ug of total RNA for each sample by Illumina TruSeq Stranded Total RNA Library Prep Gold Kit (Illumina, Inc., San Diego, CA, USA, #20020599). The first step in the workflow involves removing the rRNA in the total RNA. Following this step, the remaining mRNA is fragmented into small pieces using divalent cations under elevated temperature. The cleaved RNA fragments are copied into first strand cDNA using SuperScript II reverse transcriptase (Invitrogen, #18064014) and random primers.

This is followed by second strand cDNA synthesis using DNA Polymerase I, RNase H and dUTP. These cDNA fragments then go through an end repair process, the addition of a single 'A' base, and then ligation of the adapters. The products are then purified and enriched with PCR to create the final cDNA library.

The libraries were quantified using KAPA Library Quantification kits for Illumina Sequencing platforms according to the qPCR Quantification Protocol Guide (KAPA BIOSYSTEMS, #KK4854) and qualified using the TapeStation D1000 ScreenTape (Agilent Technologies, # 5067-5582). Indexed libraries were then submitted to an Illumina NovaSeqX (Illumina, Inc., San Diego, CA, USA), and the paired-end (2×100 bp) sequencing was performed by the Macrogen Incorporated.

SUPPLEMENTARY FIGURE LEGENDS

Supplementary Figure 1. Schematic figure about KRT14cre-AIMP1 conditional transgenic (cTG) mice and AIMP1 expression (a) A schematic diagram of the skin-specific AIMP1 inducible mouse. **(b-c)** Validation of cTG by genotyping and IF staining of AIMP1.

Supplementary Figure 2. Identification of AIMP1 function with AIMP1-induced mice. (a) A schematic diagram of systemic-human AIMP1-inducible mouse model, CAG-rtTA; tetO-hAIMP1 (iTG). **(b)** Characterization of iTG mice by WB and IF staining analysis. AIMP1 levels between the WT and iTG mice were compared by WB in the indicated organs. Induction of HA-tagged AIMP1 was determined by IF staining with an anti-HA antibody. **(c)** The experimental design for analysis of hair growth. WT and iTG mice were clipped at postnatal 49 days. 1.6 mg/mL of doxycycline (DOX) contained water was supplied at the same day. **(d)** Mouse images of WT (con) and AIMP1 iTG. Images were obtained at PND 76. **(e)** H&E stained tissue images in WT and AIMP1 iTG mice.

Supplementary Figure 3. AIMP1 peptide enhanced hair growth. (a) Alexa 647-conjugated TN41 was applied to the dorsal skin of mice. Skin samples were collected at the indicated time points and fluorescence images were captured. Red: Alexa 647 signal, Blue: DAPI. **(b)** IF images with Ki67 from TN41-treated and non-treated regions at 20 days after clipping. The scale bars indicate 100 μm (**a**), 20 μm (**b**),

Supplementary Figure 4. Schematic diagram of co-cultivation with HFSC and DP cells

Supply Fig 1: Schematic figure about KRT14cre-AIMP1 conditional transgenic (cTG) mice and AIMP1 expression

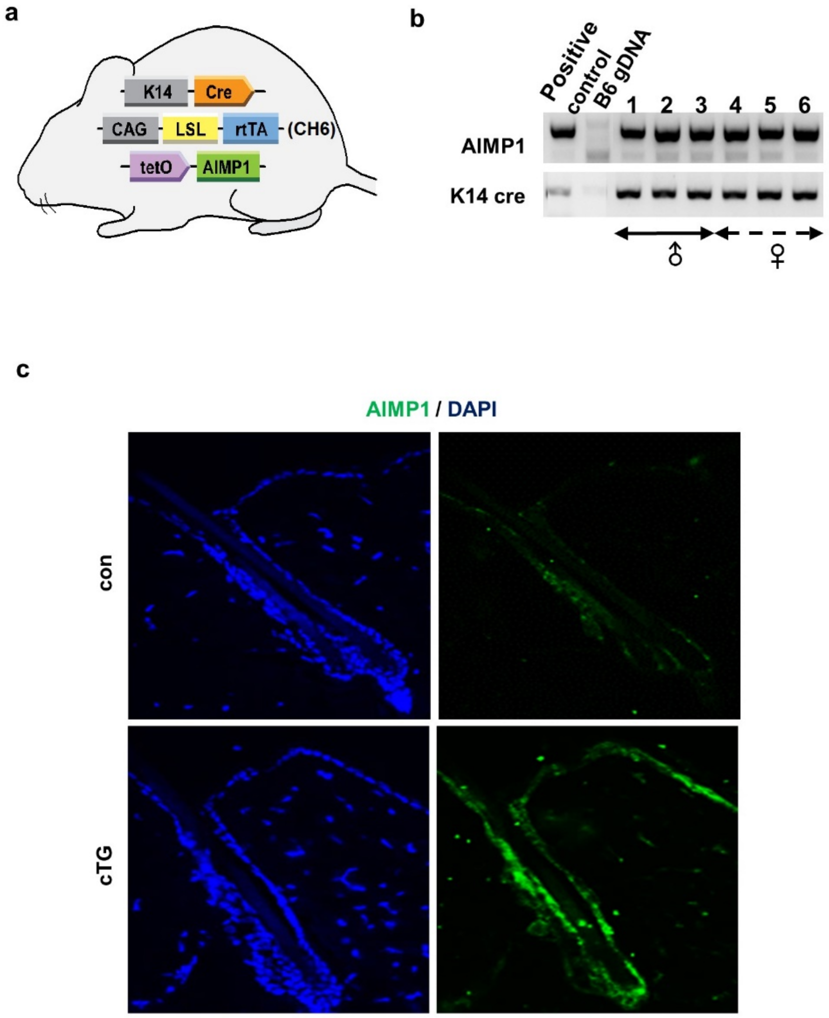

Supply. Fig 2: Identification of AIMP1 function with induced mice

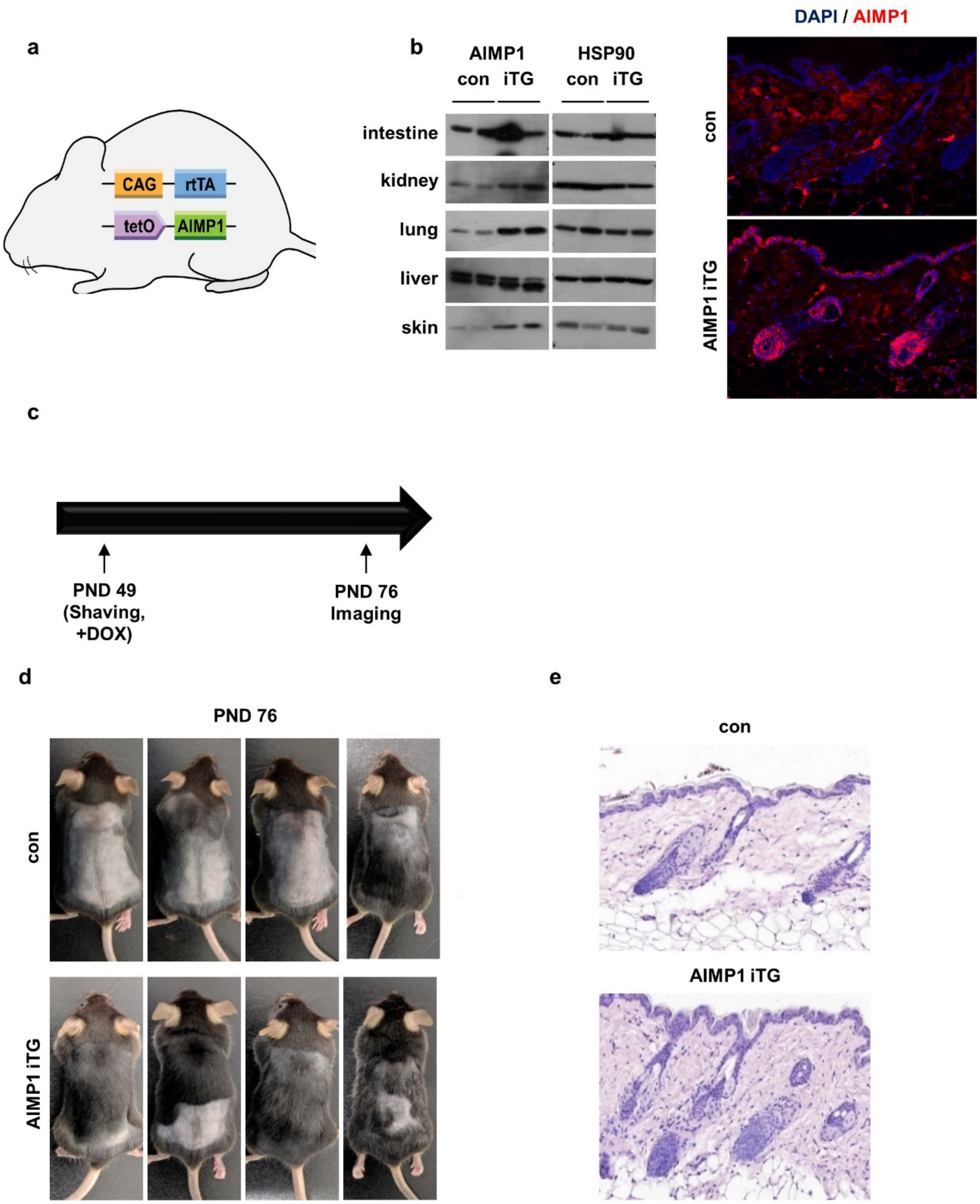

Supply Fig 3: AIMP1 peptide enhanced hair growth

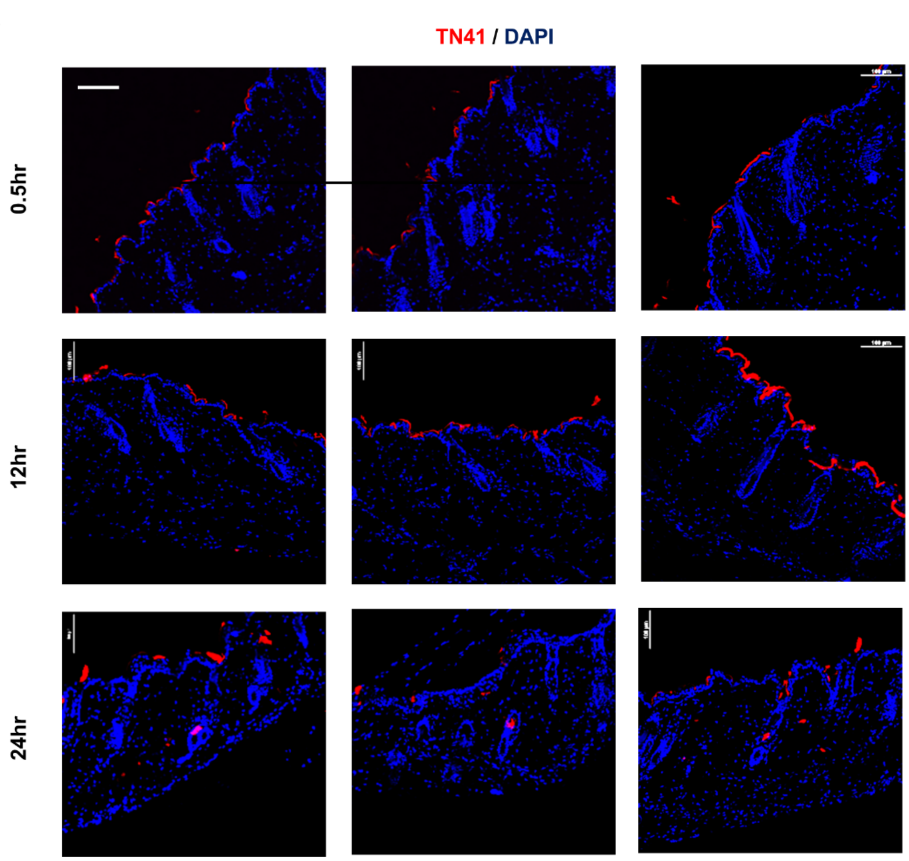

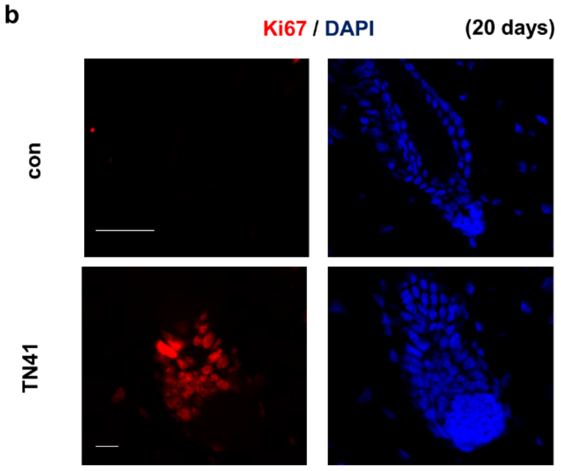

Supply Fig 4: schematic diagram of Co-cultivation with HFSC and DP cells

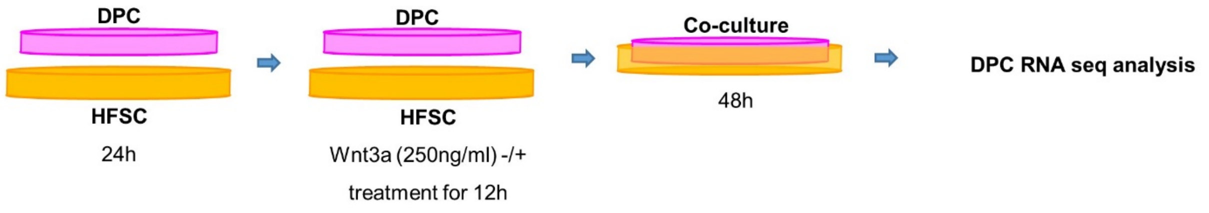

Supplement: Supplementary file 1 — Supplementary materials and methods, figures. [file ijbsv20p5764s1.pdf]
